# A Concept Analysis of Change Fatigue Among Nurses Based on Walker and Avant's Method

**DOI:** 10.1155/jonm/8413242

**Published:** 2024-11-22

**Authors:** Songmei Cao, Jingxi Lin, Yiqing Liang, Yuan Qin

**Affiliations:** Department of Nursing, Affiliated Hospital of Jiangsu University, Zhenjiang 212000, Jiangsu, China

**Keywords:** change fatigue, concept analysis, nurses, organizational change

## Abstract

**Background:** Change is prevalent in nursing environments and often leads to change fatigue among nurses while aiming to improve service quality and efficiency. Change fatigue is a significant stressor affecting nurses' work and psychology, and it is a crucial factor influencing organizational change. However, the concept of change fatigue among nurses has not yet been clearly defined or analyzed.

**Aim:** This analysis aims to differentiate, clarify, and clearly identify the specific concept of change fatigue among nurses, which will provide nursing administrators and researchers with a comprehensive understanding of the concept of change fatigue among nurses, ultimately facilitating relevant measurements and interventions.

**Methods:** This study employed Walker and Avant's concept analysis method.

**Results:** A total of 29 papers were included in the study. The four defining attributes of change fatigue among nurses were identified as nurses' exposure to constant change, exhaustion, decreased agency, and passive acceptance of change. Antecedents were categorized into nurses' personal factors and those related to the organizational environment. The consequences of change fatigue were distinguished between individual-level and organizational-level impacts.

**Conclusions:** This study provides a clearer understanding of the concept of change fatigue among nurses by outlining its antecedents, attributes, and consequences. An operational definition and conceptual understanding of change fatigue will aid future research in developing effective prevention strategies.

## 1. Introduction

Organizational change occurs frequently in the healthcare industry, encompassing innovations in new technologies, workflow adjustments, and infrastructure improvements [1]. These rapid and continuous changes aim to enhance health services and meet evolving needs and expectations [2]. However, such changes can have adverse effects on healthcare providers. They may directly impact employees' work environments, leading to organizational instability, negatively affecting employee health and psychosocial well-being, and contributing to dissatisfaction, burnout, and anxiety [3, 4]. Additionally, these changes can diminish employees' sense of belonging and loyalty to the organization [5], resulting in increased rates of sick leave and turnover [6]. Research on employees' emotional responses to change is crucial. Bernerth's research highlights that sustained change can lead to emotional exhaustion, also known as change fatigue [7], a concept initially identified in the management discipline [8] and recognized as a significant factor influencing organizational change [9].

Change fatigue is prevalent in healthcare organizations due to the frequent and accelerating pace of change [10]. Nurses, as key members of the healthcare workforce, experience frequent changes in healthcare settings. Research by Gee et al. indicates that nurses encounter change fatigue, making it a common phenomenon [11]. Recent research has increasingly focused on change fatigue within the nursing discipline. Several studies have employed qualitative methods to explore nursing staff perceptions and experiences of rapid and sustained change [12–14]. For instance, McMillan's interviews with 14 nurses revealed that they experience significant change fatigue similar to that described in the non-nursing literature [15]. Quantitative research has also examined change fatigue among nurses. Brown et al. reported moderate levels of change fatigue among 521 nurses in acute care settings [16]. Camilleri found that large-scale changes contributed to high levels of change fatigue in nurses [1]. Factors influencing change fatigue among nurses have been investigated in several studies, including the frequency of change, hospital size [16], intensity of nursing work [17], organizational adaptive reserve [18], and personal resilience [16]. Research has shown that increased change fatigue among nurses correlates with decreased organizational commitment, job satisfaction, and a higher tendency toward burnout and turnover [19, 20]. Additionally, nurses' mental health is adversely affected, with higher levels of change fatigue significantly increasing anxiety and depression [21]. Recent reviews have enhanced the understanding of change fatigue's impact on nurses and the nursing profession, highlighting the need for strategies to prevent and address change fatigue [22].

Fatigue is a psychological state commonly experienced by individuals in healthcare settings. This sense of fatigue affects both physical and mental well-being, disrupting work performance and emotional health. Various types of fatigue have specific causes and effects. For instance, alarm fatigue results from prolonged exposure to numerous clinical alarms, leading to sensory overload and desensitization [23]. Compassion fatigue arises from prolonged empathy and assistance to others, resulting in emotional numbness due to the negative emotions of those being helped [24]. Occupational fatigue is caused by excessive demands and pressures related to work tasks, environment, and scheduling [25]. Change fatigue refers to the fatigue and resistance experienced by individuals or groups due to continuous changes [7, 9]. Change fatigue and other types of fatigue constitute the fatigue phenomena individuals may experience in healthcare settings. However, unlike other types of fatigue, it primarily stems from the uncertainty and pressure associated with organizational change.

Although “change fatigue” is prevalent among nurses and has been researched, it originates from the management discipline [21]. Research on change fatigue within the healthcare field is still relatively scarce, with many ambiguities regarding its defining attributes, antecedents, and consequences. As a relatively new concept in the healthcare industry, there is no universally accepted definition within the nursing discipline [22]. Studying change fatigue is crucial not only for theoretical advancements but also for informing organizational change practices, facilitating the smooth implementation of changes, and supporting long-term organizational development. Therefore, exploring this concept is essential for enhancing researchers' understanding of change fatigue among nurses and addressing the ongoing challenges posed by change.

## 2. Methods

### 2.1. Concept Analysis Method

Walker and Avant's concept analysis method is widely used in research to address concepts that suffer from ambiguity and lack of clear definitions. This method provides precise operational definitions that contribute to a fundamental understanding of a concept's core characteristics and enhances its application [26].

The eight-step process of Walker and Avant's method includes the following: (1) selecting a concept; (2) determining the purpose of the analysis; (3) identifying all uses of the concept; (4) determining the defining attributes; (5) constructing a model case; (6) constructing other cases; (7) identifying antecedents and consequences; and (8) identifying empirical references [26].

### 2.2. Data Source

Along with consulting dictionary resources for common definitions, we also searched empirical literature to better understand the concept of change fatigue among nurses. Keywords such as “change fatigue,” “organizational change,” and “nurs⁣^∗^” were identified based on the literature review. A Boolean search strategy combining “and” and “or” was used to search the CINAHL, Embase, ProQuest, PubMed, Scopus, Web of Science, China National Knowledge Infrastructure (CNKI), and Wan Fang Database for studies from inception to June 2023, and relevant references were tracked (Table 1).

### 2.3. Screening Method

Primary literature was independently and rigorously screened by two researchers according to inclusion and exclusion criteria, removing duplicates, articles not in English or Chinese, and irrelevant articles based on titles or abstracts. All studies contributing to the understanding of the concept of change fatigue, regardless of research methodology quality, were included. Studies were included if they referred to at least one of the following aspects of change fatigue: definition, attributes, antecedents, consequences, and empirical references.

## 3. Results

**Figure 1:**
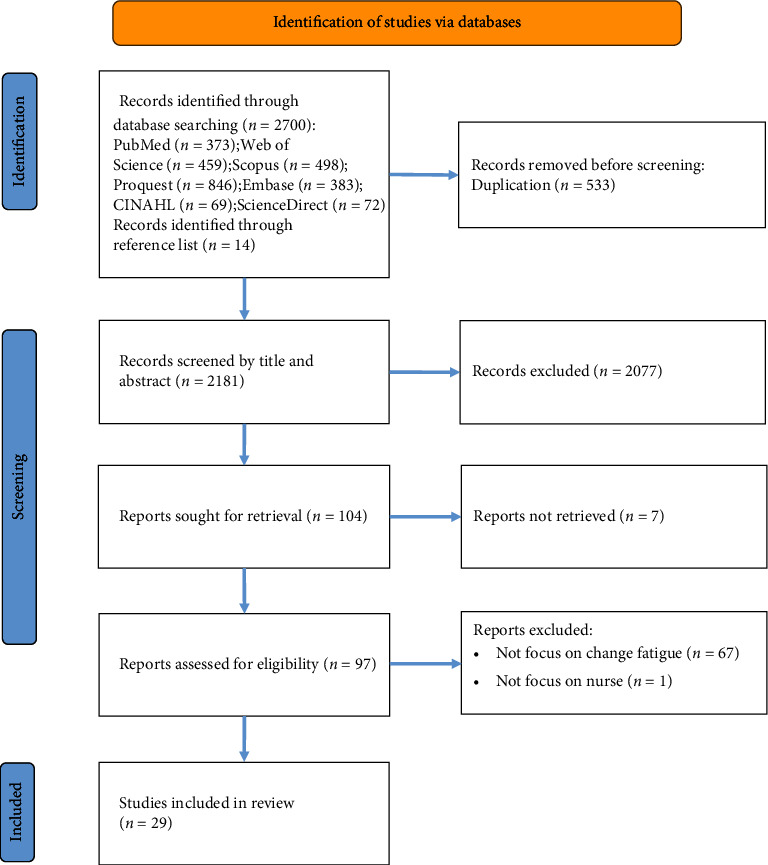
Flow diagram of the study selection process.

This search yielded a total of 2700 articles, and 14 relevant references were tracked. After removing 533 duplicates, 2181 records remained. A total of 104 articles were retained after the initial screening of titles and abstracts (*n* = 2077). Seventy-five records were excluded for the following reasons: unavailability of full text (*n* = 7), not focus on change fatigue (*n* = 67), and population not being nurses (*n* = 1). Ultimately, 29 studies in nursing or health sciences were included and analyzed (Figure 1). All studies were published between 2002 and 2023. Of these, 21 studies were from North America (USA and Canada), 5 from Europe, 2 from Australia, and 1 from Türkiye. Regarding study types, eight were quantitative, four were qualitative, four were mixed methods, three were literature reviews, and the remaining were text and opinion pieces. Notably, Kim McMillan was the first author in four studies, and Robin Brown was involved in three studies.

### 3.1. Uses of Concept

Change fatigue comprises the terms “change” and “fatigue.” According to the Cambridge Dictionary and Oxford English Dictionary, “change” is defined as “The action or process of making or becoming different.” In the nursing field, change primarily arises from organizational needs, new evidence-based practices, or requirements from governing bodies [19]. Such change often involves organizational adjustments, alterations, or transformations in response to internal and external environmental factors [27]. “Fatigue” is defined in Webster's Dictionary as “weariness or exhaustion from labor, exertion, or stress,” encompassing both physical and emotional fatigue. Various fatigue issues frequently associated with clinical work include alarm fatigue, compassion fatigue, and working time fatigue, all of which impact care safety and practice. Change fatigue has also been recognized as a potential influencing factor [10].

The topic of change fatigue is repeatedly mentioned among employees, manifesting as passive acceptance of change, which depletes adaptive resources and leads to exhaustion and other negative outcomes [13]. There are different definitions of change fatigue. Kapping [19], citing Bernerth et al. [7], defines change fatigue as “a feeling of apathy toward the organization, passive acceptance of initiatives, powerlessness, and emotional exhaustion with limited resources such as energy, personal control, or time due to excessive organizational change.” This definition underscores the psychological impact of organizational change, with these psychological shifts often evolving gradually throughout the change process [28]. In the management discipline, Cox also emphasizes the feeling of exhaustion and the harm it brings to the change process, defining change fatigue as “the exhaustion employees experience from organizational changes, which makes the possibility of successful future changes unlikely” [9].

In the healthcare industry, change fatigue is prevalent among key participants in the change process, primarily doctors and nurses [29]. Research indicates that nurses are particularly negatively affected by change [20], as they are responsible for managing, implementing, and evaluating changes [12]. For nurses, change fatigue results from the rapid and continuous changes within healthcare institutions, a trend expected to persist and increase [10, 30]. Initially, change fatigue among nurses was broadly understood as “the overwhelming feelings of stress, exhausted, and burnout associated with rapid and continuous change in the workplace” [31]. McMillan later defined it as “feelings of apathy, ambivalence, disengaged, exhaustion, and burnout during periods of ongoing organizational change” [2]. Additionally, some scholars view change fatigue not only as a personal psychological experience but also as a deterioration in both individual and organizational adaptability [32]. Nurses experiencing change fatigue find themselves drained of energy and struggling to adapt to new changes [33]. Excessive and rapid changes can impair nurses' ability to cope, leading to increased fatigue [11, 12, 34, 35]. Change fatigue represents a cumulative effect, where simultaneous and ongoing changes undermine organizational stability and increase the risk of errors among nurses [32].

### 3.2. Defining Attributes

The core of concept analysis is to identify the defining attributes of a concept—those attributes that are most relevant and that provide insight into the concept [36]. These attributes differentiate the intended concept from similar or related concepts. Our analysis identified four defining attributes of change fatigue: exhaustion, nurses' exposure to constant change, decreased agency, and passive acceptance of change (Table 2).

Exhaustion is the most central attribute of change fatigue among nurses and represents the most intuitive experience during periods of change. In an environment of frequent organizational change, nurses face exhaustion, burnout, and feelings of being overwhelmed due to prolonged exposure to the pressures of constant change [33, 42]. This is supported by McMillan's qualitative study, which reported that nurses experienced strong feelings of exhaustion and burnout [15]. Additionally, this exhaustion is recognized as overwhelming emotional exhaustion that surpasses the individual's ability to sustain [7, 22]. Vestal also notes that the drain on resources caused by change stretches nurses to their limits, necessitating additional support or intervention to alleviate work-related stress and exhaustion [10].

Nurses' exposure to constant change is a key attribute of change fatigue. Stress in the nursing profession arises from various sources, but a crucial prerequisite for change fatigue is frequent organizational change. Many studies have found that nurses who suffer from change fatigue experience continuous and frequent change [1, 11, 16, 21, 38, 39]. The overall impact of frequent organizational changes on nurses' job responsibilities, work environment, and organizational culture forces nurses to constantly adapt to new requirements and settings, affecting both their professional and personal lives [22]. Changes typically involve two main areas: alterations in organizational structure and changes in nursing practice [6]. Organizational changes include restructuring and shifts in staffing, while changes in nursing practice involve optimizing patient treatment outcomes and enhancing the quality and efficiency of care.

Decreased agency is another key attribute of change fatigue, primarily manifesting as passive participation in change initiatives. Nurses may lose interest in change, becoming unwilling or less active in implementing new programs or processes, and simply do what is required without actively seeking improvements or innovations [42]. In addition to the weakening of intrinsic motivation, there is an increase in external control. McMillan et al. found that nurses felt that they lacked the ability to participate in decision-making during the implementation process [40]. Additionally, nurses frequently experience a sense of disempowerment during change, feeling unable to effect changes in the current situation [42]. When nurses perceive that their role in the change process is undervalued, their motivation and willingness to engage in change are diminished [20].

Passive acceptance of change represents a form of silent dissent, distinct from overt change resistance, and is more oriented toward silent conflict [18]. It is a direct response to change fatigue. Nurses experiencing change fatigue often feel dissatisfied but are reluctant to openly express their dissent [16, 31]. They may question the value and goals of the change, exhibit impatience [30, 42], or display apathy toward the change process [20, 41].

### 3.3. A Model Case

A model case exemplifies a concept by including all its defining attributes, thereby clarifying the meaning of the concept [26].

Mrs. X, aged 32, was a charge nurse with 11 years of experience at the hospital. During the COVID-19 pandemic, her department underwent multiple changes in work processes, such as the requirement to track the movements of all patients' relatives at noon and in the evening, which increased Mrs. X's workload. Concurrently, a new evidence-based practice project was implemented in the unit. However, the integration of this new practice was poorly executed. The new evidence-based practice not only demanded higher research skills but also added to the existing workload. For instance, the introduction of a new risk assessment scale required Mrs. X to evaluate patients twice upon admission. This additional task made her feel apprehensive and irritable before each procedure and led her to question the validity of the new change. Mrs. X increasingly felt that while her energies were diverted by the new changes, she still needed to meet the demands of patients and management simultaneously. As a result, Mrs. X became exhausted and dissatisfied with the changes.

In this case, change fatigue among nurses manifested through rapid and constant change over a short period, leading to exhaustion, powerlessness, and impatience and questioning the value of the change, accompanied by resistance to it.

### 3.4. A Borderline Case

A borderline case contains most of the defining attributes of a concept.

Mrs. Y, aged 31, was a nurse practitioner with 6 years of experience at the hospital. Due to hospital accreditation and the department's focus on specialty construction, her department frequently revised its routines, processes, and policies. This involved interpreting guidelines, updating specialty disease diagnoses and treatments, and conducting literature research for accreditation preparation. The increased workload and frequent overtime led to negative emotions. Despite this, Mrs. Y viewed the changes as a natural progression necessary for the hospital's successful accreditation and believed that the situation would improve once accreditation was complete. She managed her emotions and continued to support the change process through self-regulation.

This borderline case included nearly all the attributes of change fatigue: Mrs. Y experienced constant changes, developed feelings of exhaustion and powerlessness, and exhibited mild change fatigue. However, instead of remaining passive, she proactively participated in and gradually adapted to the changes, resulting in a gradual easing of her change fatigue.

### 3.5. A Contrary Case

A contrary case is one in which none of the conceptual attributes are included.

Mrs. Z, aged 34, is a charge nurse with 11 years of experience at the hospital. Despite the department undergoing numerous changes, including staffing restructuring, evidence-based practice projects, and the development of specialty areas, Mrs. Z did not experience change fatigue. The unit was well-prepared for these changes, with experienced doctors and nurses providing strong support for their implementation, enabling Mrs. Z to adapt effectively. Throughout the change process, Mrs. Z recognized the importance of these changes and believed that participating in them would contribute to the hospital's development and improve the quality of care. This perspective made her feel fulfilled and increased her sense of self-worth.

In this case, although frequent changes occurred, Mrs. Z did not develop change fatigue. Instead, she experienced a boost in self-worth as a result of the changes.

### 3.6. Antecedents

Antecedents are events or situations that must already exist before a concept can occur [26]. We reviewed the literature and classified antecedents into individual and organizational factors.

Individual factors include gender, age, and personal resilience. Brown et al. found that male nurses had higher change fatigue scores than female nurses and that personal resilience was negatively correlated with change fatigue among nurses [16]. Additionally, younger nurses were more likely to experience change fatigue than older nurses [10]. Education level also serves as a predictor of personal resilience: Higher education levels are associated with greater resilience, which indirectly affects the level of change fatigue among nurses [41].

Organizational factors include the pace of change, workload, and readiness for change.

Frequent and continuous change is a significant antecedent to the onset of change fatigue among nurses and a necessary condition for its development. Nurses work on a very tight schedule, and implementing changes within this limited time frame can disrupt their workflow, leading to burnout and fatigue. Recent empirical studies have shown that excessive organizational change makes nurses more susceptible to change fatigue [17, 19–21, 43]. Change fatigue increases with the speed, frequency, and volume of change [7]. This urgency often demands rapid adaptation of work processes, creating challenges at work and contributing significantly to psychological and emotional stress.

Both quality improvement and performance enhancement require the implementation of change. The increased workload associated with a growing number of changes can lead to heightened emotional fatigue, which is a common cause of change fatigue. During periods of change, work intensity often increases, resulting in longer shifts, more nursing duties on the wards, and additional workloads [15, 22, 38, 44]. This can lead to frequent overtime, including weekends, and necessitate adjustments in resources such as manpower, materials, and time. A study revealed that during the COVID-19 pandemic, organizational changes surged, resulting in increased overtime for nurses, with over one-third working more than 10 additional hours per week [21]. Hospital size also affects workload; larger hospitals with more beds have larger patient intakes, and some studies have indicated a relationship between hospital size and nurse change fatigue [16].

Insufficient readiness for change can also contribute to change fatigue among nurses. A lack of adequate communication and unclear change criteria during the change process can lead to concerns about the unknown or misunderstandings regarding the change plan, resulting in additional distress and stress [12, 17]. Additionally, a practice's ability to keep up with rapid change largely depends on its adaptive reserve [45]. A team with a weak adaptive reserve exhibits less resilience in implementing change and lower acceptance of it. Research has shown that insufficient adaptive reserve can lead to burnout and change fatigue [18].

### 3.7. Consequences

The consequences of a concept arise from its existence [26]. According to the literature, the consequences of change fatigue among nurses encompass both individual and organizational dimensions.

#### 3.7.1. Individual Dimension Consequences

One consequence is the change in nurses' personal behavioral intentions. Nurses often appear self-sacrificing, dedicating personal time and energy to working overtime to ensure patients receive necessary care [15]. This behavior can lead to health issues for the nurses themselves [15, 41]. Additionally, as change continues, nurses may shift their energy and resources to other activities [42] and become reluctant to engage in clinical change efforts [12]. Persistent organizational instability and disregard for nurses' concerns can increase their likelihood of leaving their jobs [2, 7, 18, 20, 35, 41].

Another consequence involves personal emotional responses. Constant adaptation to new demands and changes, without adequate time for preparation or adjustment, can exacerbate existing stress [34], leading to burnout and decreased long-term job satisfaction [16, 19, 20, 38]. It can also contribute to increased anxiety and depression [21]. When nurses' resources (e.g., time and energy) are insufficient to meet the excessive demands and expectations from superiors and patients, they are at high risk of experiencing moral distress [17]. Simultaneously, nurses may lose trust in the organization [30], resulting in decreased organizational commitment [7, 19, 20, 38] and a crisis of confidence in management [41].

#### 3.7.2. Organizational Dimension Consequences

The emergence of change fatigue among nurses can impede the implementation of practice changes. Nurses experiencing burnout may become unwilling to participate in change initiatives and may exhibit oppositional or avoidant behaviors, which can hinder change implementation and increase the risk of failure [11]. As nurses continuously encounter new changes and demands, they must adapt to new processes, technologies, and task assignments. This ongoing process can disrupt established work patterns, interfere with work habits, and lead to decreased productivity and an increased likelihood of errors [32, 34], which in turn affects the quality of care and heightens the risk of substandard care. Stability and consistency within nursing teams are crucial for providing efficient care. However, excessive change can lead to frustration and disillusionment among nurses, potentially prompting them to leave their jobs. This turnover can adversely impact organizational productivity and the team's ability to work cohesively [30].

### 3.8. Empirical Referents

The final step of concept analysis involves identifying empirical references that support the interpretation of the concept. The universal Change Fatigue Scale, developed by Bernerth et al. in 2011, was selected as the primary valid instrument for examining change fatigue [7]. Originally, the scale was part of a set of measures designed to explore the impact of multiple organizational changes on employee well-being, organizational commitment, and turnover intentions. The scale consists of six items, using a 7-point Likert scale ranging from 1 (Strongly Disagree) to 7 (Strongly Agree) to assess overall change fatigue. Higher scores indicate a greater degree of change fatigue. The scale has demonstrated good reliability and internal consistency in larger sample populations, with a Cronbach *α* of 0.85 in non-nurse populations. In 2018, Brown et al. reported a Cronbach *α* of 0.94 for the scale in a sample of 521 nurses [16]. Cox adapted this scale to five items, while still using a 7-point Likert scale [9]. However, research tools specifically designed to effectively identify change fatigue among nurses are still lacking. Existing tools often lack relevance and specificity for nurse populations, fail to encompass all attributes of nurse change fatigue, and do not fully capture the phenomenon among nurses.

### 3.9. Definition of Change Fatigue Among Nurses

Change fatigue among nurses is defined as a state of exhaustion, decreased agency, and passive acceptance of change resulting from exposure to constant change. This condition negatively impacts both the physical and mental health of nurses, as well as affects the organization as a whole.

## 4. Discussion

The purpose of organizational change is to enable the organization to adapt to a changing environment. However, change fatigue resulting from frequent change poses a major challenge to organizational change [15]. The impact of epidemics, in particular, introduces additional burdens of organizational change and psychological stress [21, 46]. This increased awareness among care managers about change fatigue highlights the need for a standardized definition of the concept. This concept analysis aims to provide a comprehensive understanding of change fatigue among nurses, benefiting both research and practice, and to draw attention to its potential impacts. Furthermore, this analysis may assist in subsequent incident reporting, risk monitoring, and intervention evaluation, ultimately exploring effective strategies for managing fatigue. Thus, this concept analysis has significant implications for research on change fatigue among nurses.

**Figure 2:**
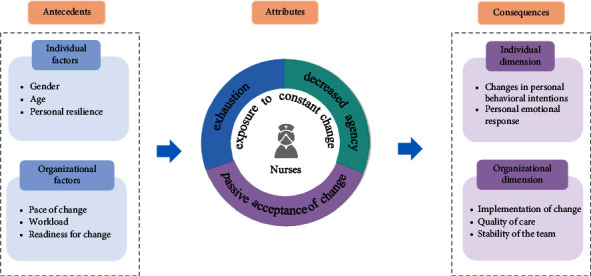
Change fatigue concept analysis diagram.

According to Walker and Avant's method [26], understanding change fatigue among nurses involves identifying its attributes, antecedents, and consequences. Change fatigue is a complex experience that arises when nurses are subjected to frequent and continuous changes, negatively affecting both their physical and mental health, as well as the organization. The concept encompasses four defining attributes: exhaustion, nurses' exposure to constant change, decreased agency, and passive acceptance of change. The literature indicates that exposure to constant change is the most critical attribute of change fatigue in clinical care environments. The attributes of exhaustion, decreased agency, and passive acceptance are the most significant manifestations of change fatigue in contexts of frequent change (Figure 2).

The antecedent analysis of this study reveals that change fatigue among nurses is influenced by both individual and organizational factors. It has been suggested that nurses and their managers might not recognize change fatigue and may consider it a normal part of the job [39]. Identifying these antecedents underscores the importance of proactively coordinating change to successfully implement new practices [47]. Our literature review identified gender, age, and personal resilience as individual factors affecting change fatigue among nurses. Recognizing these factors can help increase nursing managers' awareness of risk factors for change fatigue and identify potential causal elements. Analyzing organizational factors can aid nursing managers in better assessing and responding to environmental changes, thereby enhancing the organization's capacity for development. There is a need for decision-makers to educate and train nurses about change fatigue [43]. By thoroughly understanding the causes of nurse change fatigue and utilizing these insights to develop personalized and comprehensive strategies, better evaluation and prevention of change fatigue among nurses can be achieved.

After analyzing the consequences of change fatigue, it is evident that it is critical to the successful implementation of organizational change. Nurses, being a crucial part of the healthcare team, directly interact with patients and provide care during the implementation of changes within the healthcare team. However, change fatigue negatively impacts nurses, leading to burnout and stress, as reported in various studies [18, 20, 21]. Additionally, individual emotional experiences affect job satisfaction, which is a key factor in recruiting and retaining nursing talent [48]. Increased nurse turnover undermines organizational stability and development [30]. Therefore, timely and effective intervention and management of change fatigue among nurses are essential to address the challenges posed by subsequent changes. Establishing positive responses to these challenges can help nurses become more resilient and reassess their competence and value. Enhancing job satisfaction and increasing willingness for change can mitigate the difficulties associated with transformation. To some extent, the alignment and implementation of change are also influenced by the feedback from the consequences of change fatigue.

Despite the significant implications of change fatigue for nurses in the context of organizational change, this concept is rarely applied in clinical nursing settings. This may be due to the reliance on universal scales from management disciplines to measure change fatigue [7]. Further research is needed to develop assessment tools specifically tailored for nurses to recognize signs of change fatigue and identify strategies to proactively reduce its occurrence. The development of such tools should consider all defining attributes of change fatigue and not be limited to a single aspect. Additionally, factors related to various nursing environments, such as community nursing, nursing homes, and home care, should be incorporated. Specific measurement scales for different types of changes in nursing practice are also necessary due to the complexity of change in clinical settings.

Although specific interventions to prevent and mitigate change fatigue among nurses are still limited, many studies emphasize the importance of active support from nursing managers. Decision-makers are urged to organize and manage change programs effectively to reduce the incidence of change fatigue [22]. Research also suggests strategies such as providing information and training support to help nurses understand and actively engage with changes [43]. Supporting open communication can reveal change fatigue and promote the sustainable development of change initiatives [49].

Change fatigue among nurses remains a complex issue, and current research is insufficient for a comprehensive and systematic assessment of the influence and interrelationships among its antecedents. Future research needs to explore the impacts of factors such as the work environment, frequency of changes, and individual psychological resilience. The interaction of these factors may reveal the underlying causes of change fatigue. As highlighted by this conceptual analysis, no single manifestation fully captures nurse change fatigue, emphasizing the need to consider the overall picture and develop comprehensive assessment scales. Additionally, reducing nurse change fatigue is crucial for facilitating effective change [9]. Therefore, designing preventive strategies, exploring proactive interventions, and evaluating their effectiveness are important areas for further research. The study of change fatigue should involve multidisciplinary collaboration, advocating for interdisciplinary and cross-institutional research integrating knowledge from psychology, management, sociology, and other fields for a thorough investigation.

While this study provides valuable insights into change fatigue among nurses, it has limitations. Firstly, the article's focus on English and Chinese sources may limit the scope of the research, potentially overlooking important perspectives. Secondly, the analysis of attributes and definitions may be subjective, as different individuals might have varying interpretations of change fatigue, introducing potential bias. Additionally, the variability of concept definitions and the evolving nature of healthcare policies and practices may not be fully reflected, potentially limiting the study's temporal relevance. This suggests a need for new research to analyze the evolution of change fatigue over time and in response to societal changes.

## 5. Implications for Nursing Management

By analyzing the concept of change fatigue among nurses, this study clarifies its significance and has broad implications for nursing management. First, the concept analysis aids nursing managers in developing a unified understanding of change fatigue, which is crucial for guiding subsequent research and avoiding confusion in findings. This clarity helps reduce uncertainty and challenges in nursing management, ultimately enhancing efficiency and quality. Second, analyzing the antecedents and consequences, along with specific cases, offers insights into the risk factors for change fatigue. This information provides valuable guidance for managers in developing more effective and practical change programs, thus improving the success rate of organizational changes. Finally, understanding change fatigue among nurses introduces new ideas and methods for innovating nursing management. It opens the door to developing new management models, technologies, and tools designed to reduce change fatigue and support sustainable change.

## Figures and Tables

**Table 1:** Search strategy.

Restrictions	Contents
Research topic	Concept analysis of change fatigue in nursing

Search strategy	“Change fatigue” OR “organizational change” AND “nurs⁣^∗^”

Limits and type of material required	From inception to June 2023
	English language or Chinese language
	Full text

Databases and resources to be searched for relevant	CINAHL
	Embase
	ProQuest
	PubMed
	ScienceDirect
	Scopus
	Web of Science
	China National Knowledge Infrastructure (CNKI)
	Wan Fang Data

**Table 2:** Defining attributes.

Defining attributes	Sources
Nurses' exposure to constant change • Changes in organizational structure • Changes in nursing practice	[1, 2, 10–12, 15–17, 19–22, 29, 31–35, 37–41]

Exhaustion • Beyond one's capacity • Need additional support or interventions • Lack of energy	[2, 10, 12, 13, 15–19, 22, 31, 33–35, 38, 41, 42]

Decreased agency • Feeling more passive • The lack of involvement in decision-making	[2, 15–17, 19, 20, 22, 31, 33, 40–42]

Passive acceptance of change • Questioning the value and objectives of the change • Showing impatience	[2, 16, 18, 22, 30, 35, 41, 42]

## Data Availability

The data used to support the findings of this study are available from the corresponding author upon reasonable request.
